# Did avian-like pulmonary anatomy increase the susceptibility of dinosaurs to fungal diseases? Extending the “fungal infection mammalian selection” hypothesis

**DOI:** 10.1128/mbio.00521-26

**Published:** 2026-06-18

**Authors:** Isabel A. Jimenez, Arturo Casadevall

**Affiliations:** 1 Department of Molecular and Comparative Pathobiology, Johns Hopkins School of Medicine 1500 , Baltimore, Maryland, USA; 2 Department of Molecular Microbiology and Immunology, Johns Hopkins Bloomberg School of Public Health 25802 , Baltimore, Maryland, USA; Instituto Carlos Chagas, Curitiba, Brazil

**Keywords:** paleomicrobiology, comparative anatomy, dinosaurs, veterinary medicine, cretaceous-paleogene, mycology, fungal disease, evolutionary biology

## Abstract

The Cretaceous-Paleogene (K-Pg) bolide impact caused the extinction of over half of all plant and animal species. The “fungal infection mammalian-selection” (FIMS) hypothesis, first proposed in 2005, suggested that a post-K-Pg fungal bloom created selective pressures that favored the survival and evolutionary radiation of warm-blooded mammals due to their resistance to fungal diseases, while cold-blooded dinosaurs were at an evolutionary disadvantage. Evidence now indicates that dinosaurs were also endotherms; hence, body temperature alone was unlikely to have predisposed all dinosaurs to fungal infection. The fossil record suggests that avian-like respiratory anatomy was present in archosaurs, the ancestors of all dinosaurs. Here, we update the FIMS hypothesis, drawing on insights from modern bird anatomy and physiology, to suggest that dinosaurs may have been more susceptible to respiratory diseases, including fungal infections, perhaps sufficiently to tip the scales away from a second dinosaur age in a world replete with fungal spores.

## OPINION/HYPOTHESIS

## GRADUALISM TO CATASTROPHE: HISTORICAL CONTEXT OF DINOSAUR EXTINCTION

Dinosaurs have long captivated the public imagination, their legacy suspended between scientific reality and cultural mythos. The term *Dinosauria* was coined in 1842 by British paleontologist Richard Owen. By the late 19th century, the discovery of dinosaur fossils had been transformed into a fierce competition between rival paleontologists, later termed the “bone wars” (1). Wealthy patrons poured funding into paleontological expeditions and filled natural history museums with evidence of their discoveries. By the mid-20th century, dinosaurs were firmly embedded in popular culture. Yet despite their prominence in both science and public imagination, the question of what caused their disappearance remained largely unresolved. Initially, the extinction of the dinosaurs was largely believed to be a gradual process, driven by ecological and climatic changes and increased competition from mammals (2). In 1980, Alvarez et al. discovered that sediment layers at the Cretaceous-Paleogene (K-Pg) boundary were highly enriched in iridium and proposed that a bolide impact had suddenly and catastrophically ended the age of the dinosaurs (3). Their proposal was initially met with skepticism, but over the following decades, supporting geological evidence accumulated, including the identification of the Chicxulub crater in the Yucatán Peninsula as the likely impact site, and shifted paleontologists’ perspectives away from a gradual decline model (4, 5).

The K-Pg calamity is now widely accepted as the primary driver of extinction of three-quarters of the species on Earth and the end of the dinosaurs (6), through a combination of bolide impact, climate change, extreme weather, and food scarcity. When the asteroid struck, the blast triggered massive tsunamis, earthquakes, extreme atmospheric heating, wildfires, acid rain, and downpours of hot ash and debris (7). Near the impact site, intense thermal radiation and ejecta would have killed nearby organisms rapidly. In the aftermath, intense environmental conditions decimated most species of plants and animals (6). The massive atmospheric load of aerosolized dust, soot, debris, sulfur, and carbon dioxide devastated air quality and blocked sunlight, plunging the Earth into darkness and cold, a phenomenon known as impact winter (7). Trees were destroyed, taking with them the habitats of arboreal species. Most plants, phytoplankton, and algae died, unable to perform photosynthesis. Herbivores and carnivores starved, with large dinosaurs likely impacted first, given higher metabolic demands. Aquatic species overall fared better than their terrestrial counterparts but still suffered devastating losses, as ocean waters became warm, anoxic, and acidified (8, 9). Ammonites, which fed on plankton, went extinct, followed by their marine predators, mosasaurs and plesiosaurs (9). Marine crocodilians also became extinct, but freshwater turtles and crocodiles survived, perhaps buffered in inland habitats (9). Teleost fish largely survived and radiated to become the most abundant marine vertebrates (10).

## THE EVOLUTION OF THE FUNGAL INFECTION MAMMALIAN SELECTION (FMS) HYPOTHESIS

In the aftermath of the impact, why did reptiles/dinosaurs not repopulate the Earth to once again become the dominant terrestrial megafauna? Perhaps the initial destruction of dinosaurs was so complete that a diverse range of environments were left vacant, within which mammals could undergo widespread adaptive radiation. Alternatively, ecosystems were so altered by the cataclysmic events that the environment could never again support large dinosaurs. Ecological studies demonstrate that indeed, after the K-Pg-triggered trophic cascade, forest compositions were forever altered (11), influencing the evolution of modern birds, the descendants of the only dinosaurs that survived the impact (12). But while any large dinosaurs that survived the initial cataclysm likely starved quickly given the disrupted biosphere, small mammals as well as small terrestrial dinosaurs with fossorial behavior could have gone to ground, seeking cover from still-volatile surface conditions in subterranean burrows or beneath hardy ferns (13). Yet only a small subset of small dinosaurs survived into the modern era to become the species we now know as birds. Why did small mammals ultimately win the evolutionary race over most small dinosaurs?

In the early 2000s, Dr. Casadevall felt that the dominance of mammals was still difficult to explain (14). This was at a time when dinosaurs were commonly thought to be ectothermic, or “cold-blooded,” relying on their environment to determine their temperature, like reptiles. Given the resource-strapped landscape in the wake of the impact, it seemed intuitive that reptiles would better withstand food scarcity and temperature fluctuations through behavioral thermoregulation. The higher metabolic rate of mammals required increased food and water. In the absence of consistent resources, mammals cannot sustain prolonged metabolic heat production and become prone to hypothermia. Insufficient water also leads to difficulties withstanding heat via evaporative cooling. In this regard, mammals could have been at a disadvantage.

The “fungal infection mammalian selection” (FIMS) hypothesis proposed that despite its high metabolic cost, the endothermy of mammals was a benefit, not a hindrance, in the post-K-Pg world because mammals were better able to deal with fungi (14–16). After the impact, decaying organic matter was replete, and the fossil record supports the presence of a fungal bloom (17, 18). FIMS hypothesized that for any dinosaurs managing to survive the K-Pg event, a post-disaster fungal bloom could have drastically increased selective pressures against dinosaurs, contributing to the extinction of all but the lineage that led to modern birds. In contrast, a greater proportion of small mammals survived, proliferated, and provided larger founder populations that eventually led to the great mammalian radiation, ushering the age of mammals. In this regard, mammals are remarkably resilient against fungi. Mammalian endothermy provides a thermal barrier that protects against most colonization, and mammalian immunity is also adept at preventing fungal infection (19). Conversely, ectothermic vertebrates, such as reptiles and amphibians, are highly susceptible to fungal diseases, as evidenced by mycoses currently ravaging frogs and salamanders (20), among others. Fungal pneumonia due to *Aspergillus* also occurs in crocodilians (21, 22). In 2020, the FIMS hypothesis was further modified to consider other advantages of mammals over reptiles, including the ability to forage in the long winter after the bolide impact (16).

**Fig 1:**
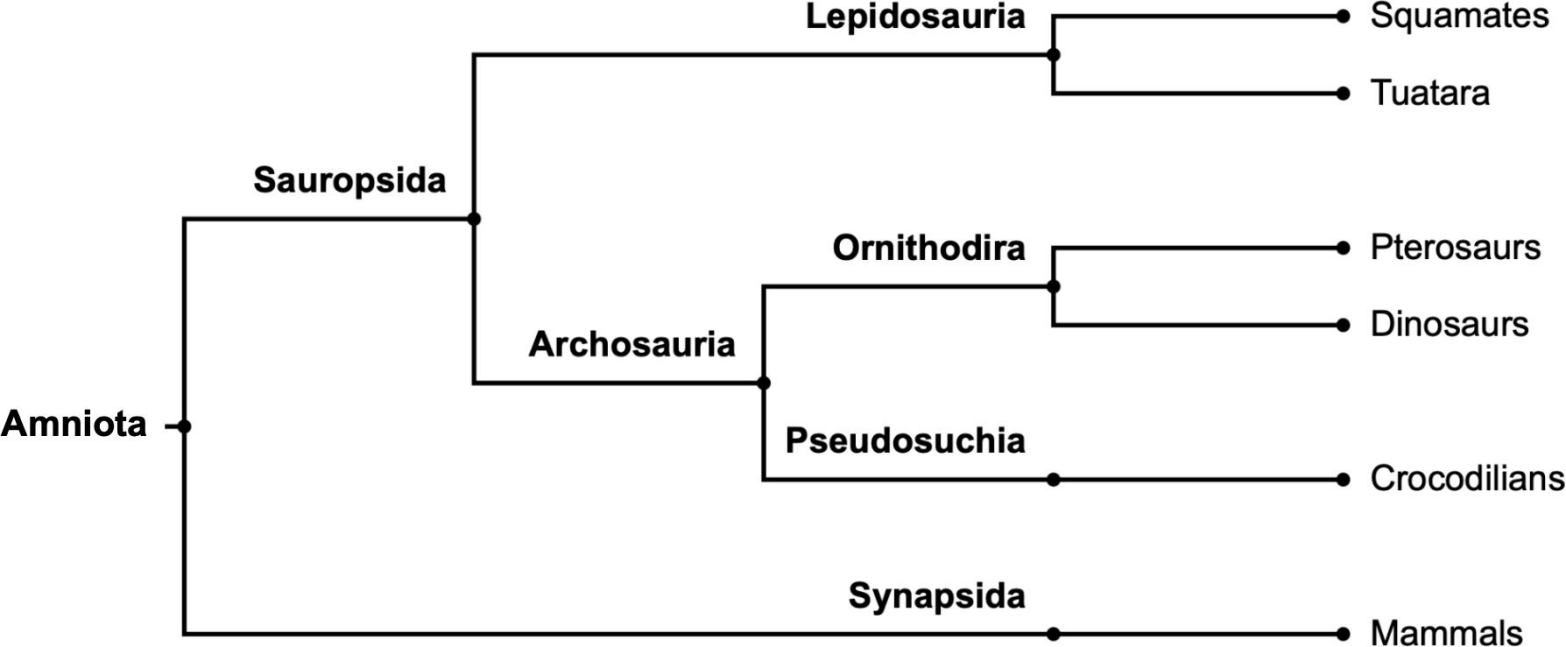
Cladogram showing the relationship between the clade Synapsida, which includes mammals, and the clade Sauropsida, which includes Lepidosauria (snakes, lizards, and tuatara) and Archosauria (pterosaurs, dinosaurs, and crocodilians). Archosaurs, the ancestors of dinosaurs, were thought to have been endothermic. All modern birds are descendants of one lineage of theropod dinosaurs that survived the Cretaceous-Paleogene extinction event. Created with Cladogram Maker (https://cladogrammaker.com/).

The original FIMS hypothesis hinged on a dichotomy of warm mammals and cold, reptilian dinosaurs. In the intervening two decades since FIMS was first articulated, mounting paleontological evidence suggests most dinosaurs were warm (23–27), having inherited endothermy from their common archosaur ancestor (Fig. 1) (24), although some controversy still remains as to exactly how endothermic dinosaurs truly were compared to modern mammals. For an excellent review, the reader is directed to Benton (28). In summary, the presence of dinosaurs in polar regions points to an ability to maintain internal body heat. The presence of feathers in dinosaurs, which evolved long before flight, suggests a need to conserve internal body heat via insulation. The erect posture of dinosaurs further indicates they maintained activity levels likely only possible via endothermy. In addition, isotopic and metabolic analyses of fossilized bones and histology demonstrating rapid bone growth all point towards high metabolic rates in dinosaurs, in some cases as high as birds and perhaps higher than modern mammals. Thus, endothermy appears to be a common trait for most dinosaurs present at the time of the K-Pg event, except for certain species of large herbivorous dinosaurs, such as *Triceratops* and *Stegosaurus* (24), as well as crocodilians (29) that evolutionarily reverted to ectothermy.

**Fig 2:**
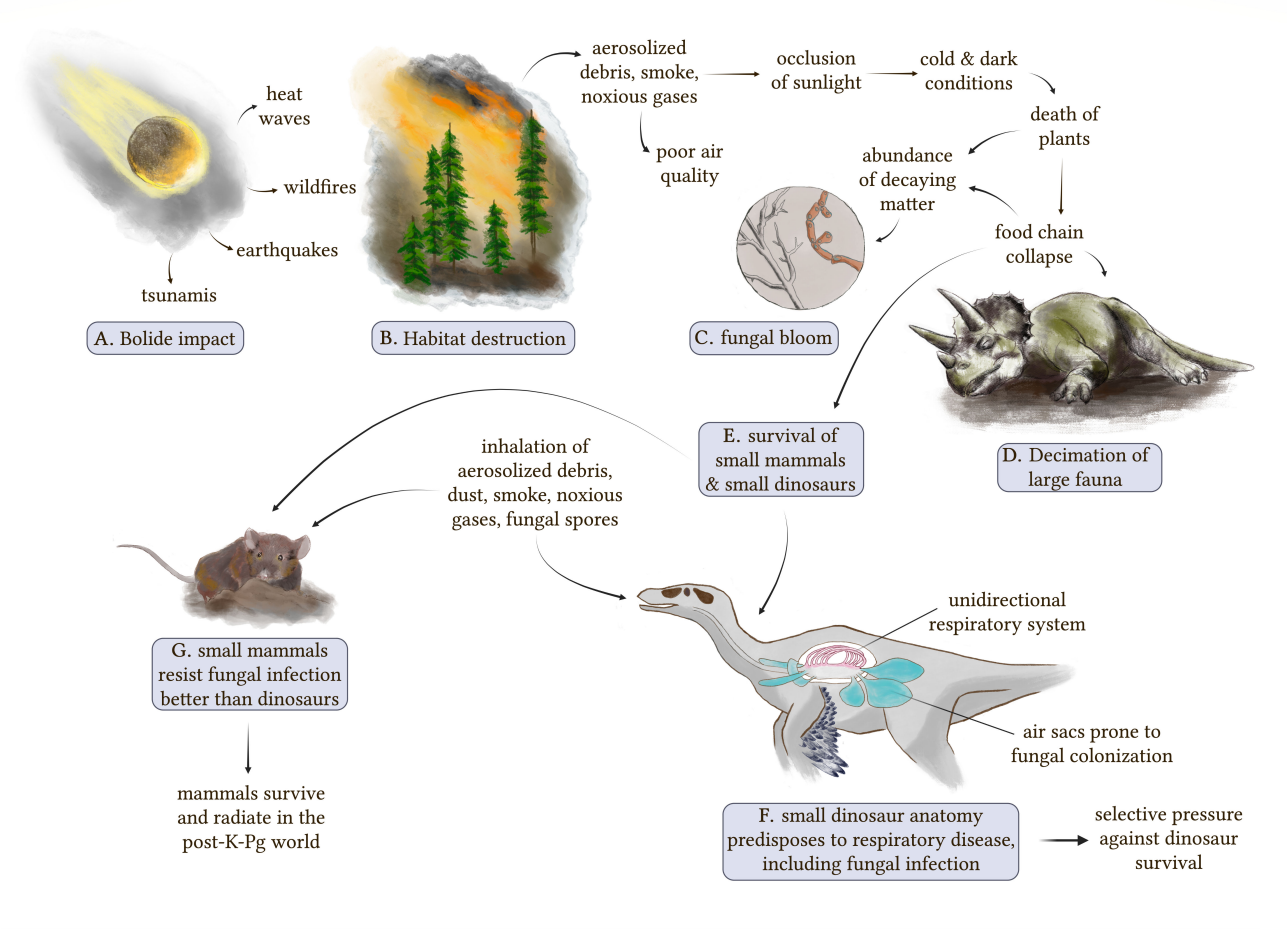
Graphical depiction of proposed factors affecting dinosaur extinction after the Cretaceous-Paleogene impact. (A) Bolide impact creates physical global destruction to the biosphere due to earthquakes, tsunamis, and rapid atmospheric heating. (B) Habitat destruction results from initial impacts and post-bolide winter, whereby dust, smoke, and soot block sunlight, reducing photosynthesis and promoting death of plants and phytoplankton. (C) Global fungal bloom occurs as fungi proliferate in damaged and decaying biosphere. (D) Large dinosaurs that survived the initial physical impacts perish due to starvation. (E) Small dinosaurs and small mammals survived by virtue of their smaller size and ability to find shelter underground or in protected sites. (F) Small dinosaur anatomy makes them more susceptible to respiratory diseases, including fungal infection, compared to small mammals. (G) As the biosphere recovers, a larger number of mammals go on to become founder populations of the mammalian radiation in the Paleogene, while small dinosaurs that survived the initial calamity could not compete. Most small dinosaurs went extinct, with the only surviving lineage being small theropods that evolved into birds. Created in BioRender (https://BioRender.com/qel8zlr).

The increasing evidence for dinosaur endothermy introduces a major gap in the FIMS hypothesis that demands reassessment. Here, we revisit the FIMS hypothesis, focusing not only on the role of body temperature in conferring resistance to fungal infection, but also on including potential anatomical differences between dinosaurs and mammals that could have predisposed the former to mycotic diseases. Even without a major core body temperature differential, could fungi still have disproportionately affected small dinosaurs over small mammals? We suggest that the avian-like respiratory system of dinosaurs would have predisposed them to fungal disease and may have contributed to their decline in the period after the K-Pg event (Fig. 2).

## DINOSAURS HAD AVIAN-LIKE RESPIRATORY ANATOMY

In mammals, contraction of the diaphragm leads to lung expansion, generating negative pressure that pulls air down the respiratory tree and into the alveoli, the sites of gas exchange. Each two-phase breath cycle thus contributes to gas exchange only after inspiration. In contrast, the fossil record shows that the respiratory anatomy of dinosaurs bears striking similarities to their closest modern relatives, birds (28). The avian respiratory system was once thought to have evolved to support flight, which comes with intense oxygen demands. However, the presence of these anatomical features in archosaurs confirms that at least the basal structure of avian-like respiratory systems evolved before flight and was a feature of most, if not all, dinosaurs at the K-Pg boundary. The major challenge in studying dinosaur respiratory biology and physiology is the lack of living specimens and reliance only on fossils. However, we can look to modern birds for clues, as specialized anatomy is often linked to function.

Avian lungs consist of a flow-through system in which air passes unidirectionally between conducting airways and air sacs, which act as holding chambers and bellows to move air back and forth across the air capillaries, where air runs countercurrent to blood, allowing gas exchange during both inhalation and exhalation. Modern birds also have a blood-gas barrier over 50% thinner and a respiratory surface at least 15% greater than mammals of a comparable size, further contributing to respiratory efficiency (30). The fossil record generally does not preserve this delicate microscopic anatomy; thus, paleontologists have yet to determine if highly efficient oxygen extraction evolved before or after the emergence of flight. In a rare specimen of fossilized lung tissue from Archaeorhynchus, an extinct beaked prehistoric bird, paleontologists identified gas exchange tissue and air capillaries that suggest an analogous oxygen exchange system to modern birds (31). Like birds, many dinosaurs also had pneumatic bones that communicated with their air sacs, conferring benefits for active, highly metabolic lifestyles (28). Pneumaticity allows the internal reinforcement of bones with strut-like architecture, providing strength for powerful muscular attachments, sustained activity, and in dinosaurs, enabling larger body sizes by maximizing structural integrity and minimizing biomechanical stress (32). The upper respiratory anatomy of birds also differs from mammals. Mammals have complex bony nasal turbinates lined with respiratory epithelium that participate in olfaction and also filter, warm, and humidify inhaled air. Mammalian turbinates have been implicated in the development of localized and often osteolytic fungal infections leading to nasal deformities (33–37). Birds have cartilage conchae that serve analogous functions, with the degree of complexity varying by taxon (38). Different dinosaur species also appear to have variable levels of turbinate development, with complex bony structures in ankylosaurs thought to participate in heat exchange for brain cooling (39) while *Velociraptor mongolensis* had no evidence of bony turbinates (40). However, since cartilage is less likely to fossilize, the potential for cartilage conchae similar to those in birds cannot be fully evaluated in dinosaur fossils. If dinosaurs did not have extensive nasal turbinate systems, then this could be an additional factor that facilitated the descent of fungal propagules into the lungs rather than the development of local sinonasal disease.

If form indicates function, dinosaurs that survived the K-Pg impact may have had respiratory systems highly susceptible to toxins, inhaled irritants, and aerosolized pathogens, just like their modern-day avian counterparts. Fungal respiratory infections are of particular clinical concern for birds that have underlying immune compromise, stress, or comorbid disease, particularly *Aspergillus* spp. and *Candida* spp., thermotolerant fungi capable of replicating at avian temperatures (41–49). Aspergillosis is the most common fungal respiratory infection in birds. Fungal infections are most commonly detected in captive birds, but also occur in wild birds, where naturally occurring underlying diseases, environmental stressors, and stress-induced immunosuppression predispose to secondary respiratory fungal infections (44, 45, 49). In cases of pulmonary and air sac aspergillosis in wild birds, particularly raptors and shorebirds, approximately 50% have comorbidities, including infections (viral, bacterial, parasitic, and other fungal) and non-infectious causes (45). The true prevalence of fungal diseases in wild birds is unknown, given that researchers and veterinarians can examine only a small subset of the population through field studies and clinical presentations at wildlife hospitals, and likely varies between taxa. Nonetheless, lower respiratory fungal infections such as aspergillosis are clinically more common in birds than in mammals (42).

If the same principles hold true for dinosaurs, what stressors in the post-K-Pg world could have increased susceptibility to opportunistic mycoses? A cold, dark environment would have placed endotherms, including dinosaurs, under a baseline degree of metabolic stress as they struggled to thermoregulate. Food scarcity could also have led to malnutrition, which may have immune sequelae. The atmosphere contained soot, smoke, and higher sulfur content. The inhalation of noxious gases, airborne debris, and other airway irritants could have caused respiratory damage in dinosaurs and pterosaurs. Respiratory mucosal barrier damage provides a portal of entry for inhaled pathogens. Air sacs, having poor vascularization and limited capacity for mucociliary clearance of inhaled particles, are sites particularly prone to fungal colonization. In addition, as the posterior air sacs receive freshly inhaled air, they tend to be cooler than core body temperature; conversely, the anterior air sacs receive warmed air as it exits the lungs. Poor air quality also leads to impaired reactive oxygen species (ROS) handling (50) and immune suppression in birds (51).

While the fossil record precludes direct analysis of dinosaur immune cells, modern-day birds may hold further clues as to the susceptibility of dinosaurs to fungal respiratory infections. The avian lung and air sacs have a relative scarcity of tissue-resident macrophages (52) and heterophils are first-responders. Heterophils are granulocytes in birds and reptiles that are similar to mammalian neutrophils but lack myeloperoxidase, and thus cannot produce a strong oxidative burst (53). Instead, heterophils are highly phagocytic and utilize antimicrobial peptides to kill pathogens. It has been suggested that heterophils could be less adept at withstanding invasion by fungal hyphae (54), although this has yet to be conclusively demonstrated.

## A SPARSE FOSSIL RECORD FOR RESPIRATORY PATHOGENS IN DINOSAURS

Does the fossil record support that dinosaurs were particularly susceptible to respiratory fungi? To date, there is only one report that proposes antemortem fungal infection in a fossilized dinosaur (55). Researchers identified air sacculitis with evidence of local progression to osteomyelitis, hypothesizing that the lesions were caused by a fungus similar to modern *Aspergillus* spp. (55). Aureliano et al. reported a number of dinosaurs that appeared to have died with active osteomyelitis, but a specific infectious etiology was not identified (56). However, the absence of fossil evidence does not necessarily confirm a lack of infection but rather highlights the limitations of the fossil record in accurately representing the spectrum of dinosaur disease. The fossil record is highly selective for animals that die in very specific circumstances: areas free of oxygen, decomposition, and destruction by scavengers, such as lakes, rivers, the ocean, or beneath volcanic ash (57). Even then, the fossil record primarily preserves calcium-rich mineralized structures, such as bones and teeth. Fungal diseases that affect soft tissues such as the lungs and brain are thus not expected to leave a fossil record. Disease processes chronic or invasive enough to lead to bony changes are more likely to fossilize, such as arthritis, gout, or neoplasia. In mammals, bone remodeling of the face and nasal passages is a more common feature of fungal infections, such as cryptococcosis, aspergillosis, and blastomycosis (33–37), but fungal diseases in birds do not tend to cause these deformities and instead present with either gastrointestinal or air sac/pulmonary disease. Fungal air sacculitis in birds may progress to involve pneumatic bones, most commonly vertebral osteomyelitis (58). However, this is not a universal feature of air sacculitis and also depends on the chronicity of the disease.

## CONSIDERATION FOR THE DINOSAUR LINEAGE THAT EVOLVED INTO BIRDS

The only dinosaurs that ultimately survived the K-Pg extinction were a lineage of small, non-arboreal, beaked and feathered theropods, their opportunistic omnivorous diet allowing them dietary flexibility; these are the ancestors of all modern birds (12, 59, 60). Despite the diversity of prehistoric avian dinosaurs present at the end of the Cretaceous, most archaic bird lineages perished during the K-Pg event (59), with toothed carnivores finding insufficient prey, tree-dwelling species losing habitats and nesting sites to earthquakes and wildfires, and potentially some falling prey to fungal infections as we here suggest. The reader may note an apparent incongruency in this update to FIMS, namely that the dinosaur ancestors of birds would have had the same respiratory system here proposed as a variable that increased small dinosaur susceptibility to fungi. The fossil record is insufficient to determine why small theropod dinosaurs prevailed over any other dinosaur species. Perhaps they were slightly warmer than other dinosaurs (24), just as modern birds have significantly higher core body temperatures than most mammals, giving them an edge against infection with fungal spores. In this regard, we note that the susceptibility of kiwis to disseminated cryptococcosis has been attributed to lower body temperatures relative to other birds (61). However, endothermic temperatures alone, while protective against most fungal species, are not enough to entirely protect modern birds against fungal disease. Susceptibility to fungal infection is multifactorial. Healthy birds, at least transiently, carry a “lung mycobiome” influenced by host life history (62); however, these include fungi that can become pathogenic if respiratory health is compromised. Perhaps theropod dinosaurs were just as susceptible to fungal disease as other dinosaurs and suffered some population declines, but one lineage was ultimately resilient enough in other aspects to survive and persist. The survivorship of avian theropods across the K-Pg boundary also likely reflects the combined effects of very small body size, feathered insulation, ecological flexibility, and generalist diets.

## CONCLUSION

The fungal bloom at the end of the Cretaceous Period that was first reported in New Zealand (59) has now been found in North America (18), suggesting that it was a global event, very likely to have had a major effect on the recovery of the biosphere after the Chicxulub bolide impact. Recent evidence that the Deccan volcanic event preceding the bolide impact was also accompanied by a fungal bloom (18) implies a fungal-rich environment in the late Cretaceous and early Paleogene, increasing the possibility that fungal pressure on the biosphere was an additional facet of that calamity.

The original impetus for the FIMS hypothesis was an attempt to provide some explanation for the paradoxical dominance of mammals in the Paleogene, given their significantly greater energy requirements. In this regard, mammals require anywhere from 100 to 1,000 times more metabolic energy than reptiles (63), creating a mystery for how the energetically expensive mammalian lifestyle prevailed to usher the age of mammals after the end-Cretaceous calamity. We imagine that the physical destruction that accompanied the Chicxulub bolide impact, and the trophic cascade and biosphere disruption that followed, led to the direct extinction of carnivorous dinosaurs at the top of the food chain and herbivores that lost their food sources. However, there were still small dinosaurs that presumably could have survived the initial stages following the bolide-mediated devastation. If these small dinosaurs were also endothermic, then one must posit additional mechanisms for their extinction compared to surviving small mammals. Here, we extend the FIMS hypothesis by incorporating aspects of avian respiratory anatomy and physiology to identify potential vulnerabilities to fungi that may have existed in dinosaurs. We suggest that fungal infection may have provided sufficient selective pressure against small dinosaurs to tip the scales towards the survival of larger numbers of mammals than then became the founder groups for the mammalian radiation that followed. As is evident from this essay, the FIMS hypothesis can accommodate new information and will inevitably require future revisions, as we learn more about dinosaurs, mammals, the climate, ancient fungi, and prior planetary catastrophes.
